# Characterization of Full-Length and Truncated Recombinant κ-Carrageenase Expressed in *Pichia pastoris*

**DOI:** 10.3389/fmicb.2017.01544

**Published:** 2017-08-15

**Authors:** Yuan Yu, Zhemin Liu, Min Yang, Meng Chen, Zhihan Wei, Lixia Shi, Li Li, Haijin Mou

**Affiliations:** College of Food Science and Engineering, Ocean University of China Qingdao, China

**Keywords:** κ-carrageenase, *Pichia pastoris* expression system, gene truncation, heterologous expression, enzymatic characterization

## Abstract

κ-Carrageenase belongs to glycoside hydrolase family 16 and cleaves the β-(1→4) linkages of κ-carrageenan. In this study, genes encoding the full-length (*cgkZ*), Por secretion tail-truncated (*cgkZ*Δ*Pst*) and carbohydrate binding domain-truncated (*cgkZ*Δ*CBM*) κ-carrageenase proteins were expressed in *Pichia pastoris*. The copy numbers of gene *cgkZ*, *cgkZ*Δ*Pst* and *cgkZ*Δ*CBM* were 7, 7 and 6, respectively. The enzymatic activities of recombinant enzymes cgkZ, cgkZΔPst and cgkZΔCBM reached 4.68, 5.70, and 3.02 U/mL, respectively, after 120 h of shake flask fermentation at 22°C and pH 6 in the presence of 1 % (v/v) methanol. The molecular weights of recombinant cgkZ, cgkZΔPst, and cgkZΔCBM were approximately 65, 45, and 40 kDa; their K_m_ values were 2.07, 1.85, and 1.04 mg/mL; and they exhibited optimal activity at 45–50°C and pH 6–7. All the recombinant enzymes were stimulated by Na^+^, Mg^2+^, Ca^2+^, and dithiothreitol. The end-products of enzymatic hydrolysis were mainly composed of κ-carrageenan tetrasaccharide and hexasaccharide. The removal of the Por secretion tail of κ-carrageenase promoted the transcription of κ-carrageenase gene, enhancing the specific activity of κ-carrageenase without significantly changing its catalytic properties. Although the transcription level of κ-carrageenase gene after the removal of the carbohydrate binding domain was relatively high, the specific activity of the recombinant enzyme significantly decreased. The comprehensive application of the *P. pastoris* expression system combined with the rational modification of genes may provide a novel approach for the heterologous expression of various marine enzymes with high activities.

## Introduction

κ-Carrageenan is an important raw material in the food industry, and it is mainly used as a stabilizer and gelling agent (Mora-Gutierrez et al., 1998). κ-Carrageenan is a linear sulfated polysaccharide with a repeating unit composed of α-(1→3)-4-sulfated galactose (G_4S_) and β-(1→4)-(3,6) anhydrogalactose (A) (Hargreaves et al., 2013). Its degradation products, κ-carrageenan oligosaccharides, have anti-oxidant, anti-viral, anti-tumor and anti-inflammatory activities (Raman and Doble, 2015; Yu et al., 2017). Acid hydrolysis and enzymatic hydrolysis methods are typically used to prepare κ-carrageenan oligosaccharides. Compared with acid hydrolysis methods, the enzymatic method catalyzes the hydrolysis of specific glycosidic linkages on polysaccharides, and the degree of polymerization can be controlled by adjusting the hydrolysis conditions. Thus, enzymatic hydrolysis can prevent damage to the polysaccharide structure (Zhu et al., 2016; Zhang et al., 2016). Enzymatic hydrolysis is considered a mild, environmentally friendly, and sustainable method for preparing κ-carrageenan oligosaccharides.

κ-Carrageenase (EC 3.2.1.83) specifically cleaves the β-(1→4) linkages of κ-carrageenan, which belongs to glycoside hydrolase family 16 (Mou et al., 2003). Many types of bacterial genera secrete κ-carrageenase, mainly including *Alteromonas*, *Cytophaga*, *Pseudoalteromonas*, *Pseudomonas*, and *Zobellia* (Liu et al., 2011). The structure of κ-carrageenase from *Pseudoalteromonas carrageenovora* was previously described by X-ray diffraction experiments. κ-Carrageenase molecules are mainly composed of β sheets and random coils, with each β sheet containing 6–7 β chains arranged in a “tunnel-like” three-dimensional structure. The reverse stacking of β sheets contributes to the formation of “tunnel-like” catalytic sites (Michel et al., 2001).

**FIGURE 1:**
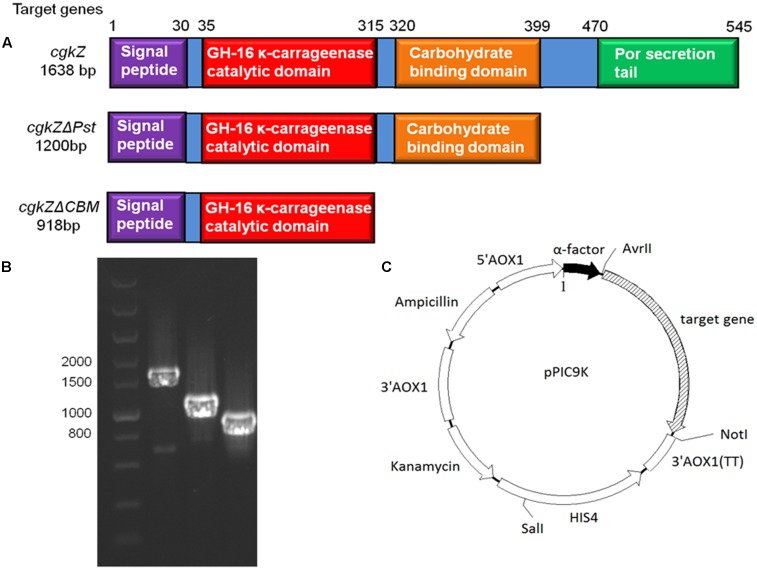
The functional area of κ-carrageenase gene and the recombinant expression vector in this study. **(A)** The functional area of *cgkZ*, *cgkZ*Δ*Pst* and *cgkZ*Δ*CBM* genes. **(B)** The PCR results of gene *cgkZ*, *cgkZ*Δ*Pst* and *cgkZ*Δ*CBM* on 1.0% agarose gel. **(C)** The vector map of recombinant pPIC9K.

The optimum temperature and pH for the activity of κ-carrageenase produced by *Zobellia* sp. ZM-2 (cgkZ) are 39°C and 6.0, respectively. This enzyme contains 12 active sites and 3 catalytic sites. The amino acid sequence of the catalytic region is E-I-D-V-V-E (Liu et al., 2013). The full-length gene encoding cgkZ is 1638 base pairs (bp) long, and the encoded protein consists of 545 amino acids (aa). The functional site of cgkZ includes the following regions (**Figure 1A**): signal peptide (1–29 aa), CH-16 κ-carrageenase catalytic domain (35–315 aa), carbohydrate binding domain (CBM, 320–399 aa), and C-terminal Por secretion tail (Pst, 470-545 aa) (NCBI).^1^ Liu et al. (2013) expressed the *cgkZ* gene in *Escherichia coli*, and found the total enzymatic activity of κ-carrageenase to be 2.98 U/mL.

*Pichia pastoris* is a eukaryotic microorganism which is suitable for high-density fermentation. Various heterologous proteins from bacteria, fungi, plants, animals, and humans have been successfully expressed in *P. pastoris* (Cereghino and Cregg, 2000; Macauley-Patrick et al., 2005; Haon et al., 2015). *P. pastoris* can perform various eukaryotic post-translational modifications including the formation of disulfide bonds and glycosylation, which can be essential for the structural stability and enzymatic activity of recombinant proteins (Haon et al., 2015; Zhang et al., 2015). In this study, the genes encoding the full-length (*cgkZ*) and truncated (*cgkZ*Δ*Pst* and *cgkZ*Δ*CBM*) κ-carrageenase proteins were expressed in *P. pastoris* to optimize the expression of recombinant κ-carrageenase and obtain recombinant κ-carrageenase having a high activity.

## Materials and Methods

### Strains, Plasmids and Media

*Zobellia* sp. ZM-2 strain (CCTCC No. M2013256) is now preserved in the China Center for Type Culture Collection (Wuhan, China). *P. pastoris* GS-115 (Mut^+^) strain and plasmid pPIC9K were purchased from Invitrogen (Carlsbad, CA, United States). Luria-Bertani medium (LB) contained 5 g/L yeast extract, 10 g/L peptone, 10 g/L NaCl, and 100 μg/mL ampicillin (20 g/L agar was added for preparing solid medium). Minimal dextrose medium (MD) contained 13.4 g/L yeast nitrogen base (YNB) without amino acids (Solarbio, Beijing, China), 20 g/L glucose, 0.4 mg/L biotin, and 20 g/L agar. Yeast extract peptone dextrose medium (YPD) contained 10 g/L yeast extract, 20 g/L peptone, and 20 g/L glucose (20 g/L agar was added for preparing solid medium). Buffered minimal glycerol medium (BMGY) contained 10 g/L yeast extract, 20 g/L peptone, 10 g/L glycerol, 3 g/L K_2_HPO_4_, and 11.8 g/L KH_2_HPO_4_.

### Gene Cloning and Recombinant Vector Construction

The complete κ-carrageenase gene (*cgkZ*) sequence was obtained from the NCBI GenBank database (accession No. KC503903). Three pairs of primers were used to amplify the genes *cgkZ*, *cgkZ*Δ*Pst* and *cgkZ*Δ*CBM* (**Table 1**). PCR was performed using *Zobellia* sp. ZM-2 genomic DNA as a template and high-fidelity *Pfu* DNA polymerase (Takara, Shiga, Japan). After the purification of the PCR products, the target genes and the vector pPIC9K were digested with *Avr*II and *Not*I (Thermo Fisher Scientific, Waltham, MA, United States). The digested products were purified by gel recycling, and the target gene and vector were ligated using T4 DNA ligase (Thermo Fisher Scientific, Waltham, MA, United States). The ligated product was transformed into DH5α competent cells. Selected positive colonies were cultured in LB medium containing 100 μg/mL ampicillin at 37°C for 12 h. The recombinant plasmids were extracted using a plasmid extraction kit (Omega, Winooski, VT, United States), and the plasmid sequences were determined by Sangon Biotech (Shanghai, China).

**Table 1:** Primers used in this study.

Primer name	Primer sequences (5′→3′)	Restriction sites
F*cgkZ*	CCGCCTAGGATGACAAAACTAAAGTTTAACGGC	*Avr* II
R*cgkZ*	ATAAGAATGCGGCCGCTTACTCCACAAGTATCTT	*Not* I
R*cgkZ*Δ*Pst*	ATAAGAATGCGGCCGCTTAAGCCGAAGTTCCGGGCG	*Not* I
R*cgkZ*Δ*CBM*	ATAAGAATGCGGCCGCTTATTCCCATACCCGAACGTAAT	*Not* I
F*GAPDH*-qPCR	GGTATTAACGGTTTCGGACGTATTG	
R*GAPDH*-qPCR	GATGTTGACAGGGTCTCTCTCTTGG	
F*cgkZ*-qPCR	TCCGTAGCCAATGGGGAAAC	
R*cgkZ*-qPCR	GGTCTTCTCCAAACCCCCTG	

*The underlined sequences represent restriction sites. F*cgkZ* and R*cgkZ* were used to amplify *cgkZ*. F*cgkZ* and R*cgkZ*Δ*Pst* were used to amplify *cgkZ*Δ*Pst*. F*cgkZ* and R*cgkZ*Δ*CBM* were used to amplify *cgkZ*Δ*CBM*. Primer F*GAPDH*-qPCR, R*GAPDH*-qPCR, F*cgkZ*-qPCR and R*cgkZ*-qPCR were used to amplify *GAPDH* gene and target genes, respectively, in real-time qPCR*.

### Electroporation and Recombinant Strain Screening

The recombinant plasmids pPIC9K-*cgkZ*, pPIC9K-*cgkZ*Δ*Pst* and pPIC9K-*cgkZ*Δ*CBM* were linearized using *Sal*I and transformed into *P. pastoris* by electroporation using a MicroPulser^TM^ electroporator (Bio-Rad, Hercules, CA, United States) at a voltage of 2 kV. After electroporation, the yeast cells were cultured in MD medium at 30°C for 3 days. Then the yeast cells were further diluted with sterile water and cultured on a YPD agar plate containing 4 mg/mL of geneticin (G418) at 30°C for another 3 days. The obtained colonies were activated in 10 mL of YPD medium at 28°C for 24 h, and then 2 mL of medium was transferred to a 1 L shaker flask containing 200 mL BMGY medium. *P. pastoris* was cultured at 28°C for 72 h with the addition of methanol (1%, v/v) every 24 h. After incubation, the culture was centrifuged and the supernatant was retained to measure κ-carrageenase activity. Enzymatic activity was measured as described by Liu et al. (2013) and protein concentration was measured using the Bradford method (Liu et al., 2011). Strains exhibiting high enzymatic activity were screened and stored at -80°C.

### Determination of Gene Copy Number

The genomic DNA of *P. pastoris* was extracted using a Yeast DNA Kit (Omega, Winooski, VT, United States). The copy number of each target gene was determined by real-time quantitative PCR using a 7900 HT real-time PCR system (Applied Biosystems, Foster City, CA, United States). A 10-fold series (10^3^–10^8^ copies) of linearized plasmids containing *cgkZ* and the endogenous gene (Glyceraldehyde 3-phosphate dehydrogenase, GAPDH), were used as templates to establish standard curves. The primers used in qPCR are listed in **Table 1**. PCR reactions were performed in a 20 μL mixture containing 10 μL of 2 × Thunderbird SYBR qPCR Mix (Toyobo, Osaka, Japan), 0.3 μM of each primer, and 50 ng of DNA template. The PCR parameters were 95°C for 60 s, followed by 40 cycles at 95°C for 15 s, 58°C for 30 s, and 72°C for 60 s. Melting curve analysis was used to determine the specificity of amplification. For each target gene, the copy numbers was calculated based on the ratio of gene copies between target gene and endogenous control.

### Optimization of Recombinant κ-Carrageenase Expression Conditions

Fermentation conditions were optimized by modulating the temperature, pH, and methanol concentration. The optimum fermentation temperature was determined by testing several temperatures (22, 24, 26, and 28°C). The optimum pH was determined in media containing 20 mM of potassium phosphate at pH 5, 6, 7, or 8. Several concentrations of methanol (0, 0.5, 1, 1.5, and 2% v/v) were evaluated to determine the optimum concentration. The recombinant enzymes were obtained by fermentation under the optimal conditions for 120 h in a 1-L shaker flask containing 200 mL of BMGY medium. Methanol (1%, v/v) was added into the fermentation culture every 24 h. The enzymatic activity was measured every 12 h.

### Determination of Transcription Levels

After incubation in shaker flask for 36 and 72 h, 1 mL of fermentation broth was used for the determination of transcription levels. Total RNA was extracted using a Yeast RNA Kit (Omega, Winooski, VT, United States). RNA was diluted with 50 μL of DEPC water and used for reverse transcription with ReverTra RT Master Mix (Toyobo, Osaka, Japan). The cDNA (25 ng) was used as a template to determine transcription levels by qPCR. Each qPCR reaction was performed as described above, with three biological replicates. The transcription level of each target gene was calculated based on the ratio of transcription levels between target mRNA and endogenous control (GAPDH).

### Purification of Recombinant κ-Carrageenase

The fermentation broth was centrifuged at 4000 × *g* for 20 min and the supernatant was concentrated by ultrafiltration using a 10 kDa membrane. Recombinant enzymes were purified by cation exchange chromatography using a CM-Sepharose Fast flow column (20 cm × 1.6 cm; GE Healthcare, Little Chalfont, United Kingdom), eluted first with 20 mM phosphate buffer (pH 6.5) for an elution volume of 30 mL, and then eluted with 20 mM phosphate buffer (pH 6.5) containing 1 M NaCl for an additional elution volume of 30 mL. Enzymatic activity and protein concentration were determined in samples obtained during purification. SDS-PAGE electrophoresis on a 12% (v/v) gel was performed to determine the molecular weights of recombinant enzymes after purification, according to the method described by Liu et al. (2011).

### Enzymatic Characterization of Recombinant κ-Carrageenase

The optimum temperature for the purified enzymes was determined by measuring their activity at pH 6 and temperatures ranging from 30 to 65°C. Thermal stability was determined as the residual activity after pre-incubating the enzymes for 0–4 h at several temperatures (40, 42.5, 45, 47.5, or 50°C). The activity of the enzymes pre-incubated at 4°C was regarded as 100%. The optimum pH for each purified enzyme was determined by measuring the enzymatic activity at 50°C in 20 mM phosphate buffer with a pH ranging from 4 to 10. To determine the effects of ions and chemical reagents, enzymatic activity was measured in solutions containing 5 mM of Cu^2+^, Ca^2+^, Zn^2+^, Mg^2+^, Fe^2+^, Fe^3+^, EDTA, SDS, dithiothreitol (DTT); 10, 50, and 100 mM of Na^+^, K^+^; 0.5% (v/v) of TritonX-100, Tween-80, methanol, and glycerol, respectively. Enzymatic activity was measured at 50°C and pH 6.0. The kinetic parameters of the purified enzymes were determined in solutions containing κ-carrageenan at concentrations between 1 and 10 mg/mL at 50°C, pH 6.0 for 5 min. The Michaelis constant (K_m_) and V_max_ were determined from Lineweaver–Burk double reciprocal plots using Origin Pro 8.0 (OriginLab, Northampton, MA, United States).

### Analysis of Hydrolysis Products of the Recombinant Enzymes

Oligosaccharides were prepared by the enzymatic hydrolysis of 10 mg/mL κ-carrageenan solution. Hydrolysis was performed in the presence of 1 mL of recombinant enzyme at 40°C for 6 h. The final product was precipitated by adding five volumes of 95% ethanol. Negative-ion electrospray ionization mass spectrometry (ESI-MS) was performed on a Q-TOF mass spectrometer (Waters, Milford, MA, United States) to analyze the oligosaccharides. The sample was dissolved in acetonitrile/water (1:1, v/v) and 5 μL of the solution containing the dissolved sample was injected for analysis with 1 mM ammonium bicarbonate /acetonitrile (1:1, v/v) as the mobile phase at a flow rate of 10 μL/min.

### Statistical Analysis

The results of fermentation optimization and enzymatic characterization experiments were analyzed by the standard deviation method, using SPSS 18.0 (IBM, New York, NY, United States). All original data represented three biological replicates. The data presented in tables are expressed as means ± standard deviations.

## Results

### Gene Cloning and Recombinant Strain Screening

The gene encoding the full-length κ-carrageenase, *cgkZ* is 1638-bp in length, whereas the genes encoding the truncated *cgkZ*Δ*Pst* and *cgkZ*Δ*CBM* are 1200 and 948-bp in length, respectively (**Figure 1A**). The results of agarose gel electrophoresis suggested that the target genes were well amplified from the genomic DNA of *Zobellia* sp. ZM-2 (**Figure 1B**). Based on the functional annotations of the *cgkZ* gene in the NCBI Genbank database, the Pst was truncated in *cgkZ*Δ*Pst* and the CBM was truncated in *cgkZ*Δ*CBM*, whereas their catalytic domains were retained. The expression vector pPIC9K used in this study contains α-factor signal sequence that can promote the secretion of recombinant proteins. The recombinant vectors pPIC9K-*cgkZ*, pPIC9K-*cgkZ*Δ*Pst* and pPIC9K-*cgkZ*Δ*CBM* were transformed into *P. pastoris* after linearization. The gene encoding histidinol dehydrogenase (*HIS4*) provides a selectable marker to isolate recombinant strains from MD plates, and the kanamycin resistance gene allows the selection of high activity colonies from YPD agar plates containing 4 mg/mL of G418 (**Figure 1C**). These strains were preserved for an analysis of gene copy number and the results are shown in **Table 2**. The qPCR results showed a linear relationship between the gene copy number and *C*_t_ value. The slope and *R*^2^ of the amplification standard curve for *cgkZ* were -3.082 and 0.995, respectively, while those for the endogenous control gene were -2.794 and 0.991, respectively. Based on the standard curve, the copy numbers of gene *cgkZ*, *cgkZ*Δ*Pst*, and *cgkZ*Δ*CBM* were approximately 7, 7 and 6, respectively.

**Table 2:** Copy numbers of target genes detected by real-time qPCR.

Target gene	*C*_t_ value (GAPDH)	*C*_t_ value (target gene)	Copy number
*cgkZ*	14.26 ± 0.17	12.24 ± 0.21	7
*cgkZ*Δ*Pst*	14.67 ± 0.12	12.66 ± 0.29	7
*cgkZ*Δ*CBM*	15.73 ± 0.35	14.14 ± 0.41	6

*C_*t*_ values are “mean ± standard deviation” from three replicates (*n* = 3)*.

### Optimization and Fermentation of Recombinant κ-Carrageenase

**FIGURE 2:**
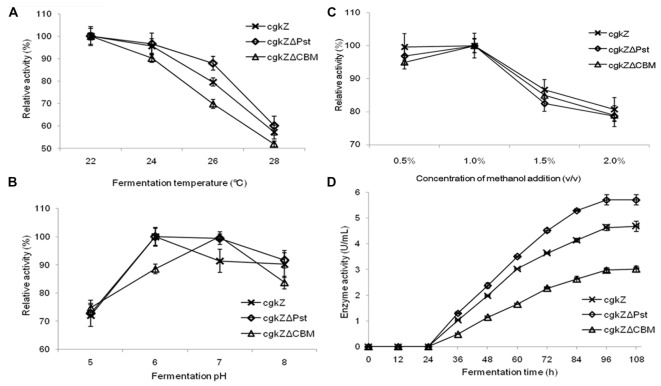
Optimization of recombinant κ-carrageenase fermentation conditions. **(A)** The effect of fermentation temperature on enzymatic activity. **(B)** The effect of fermentation pH on enzymatic activity. **(C)** The effect of methanol addition concentration on enzymatic activity. **(D)** The curve of enzymatic activity of shake flask fermentation. Error bars represent the standard deviation, *n* = 3.

In this study, the enzymatic activities of the recombinant extracellular enzymes were evaluated at four different temperatures (**Figure 2A**). The three recombinant enzymes exhibited high levels of activity below 24°C. When the fermentation temperature reached 28°C, the relative enzymatic activities decreased to 52–60%, indicating that high fermentation temperatures inhibit the secretion of recombinant enzymes. The effect of pH on recombinant enzymatic activity is shown in **Figure 2B**. The results show that the optimum fermentation pH for enzymatic activity was 6–7, which was favorable for the production of the recombinant enzymes. When the fermentation pH was 5, the relative enzymatic activity decreased to approximately 73%. The effect of methanol concentration on enzymatic activity is shown in **Figure 2C**. At methanol concentrations of 0.5–1% (v/v), large amounts of the recombinant enzymes were secreted extracellularly, and they showed high activity levels. At the methanol concentration of 1.5% (v/v), the relative enzymatic activity decreased to 82–86%.

The curve for recombinant enzyme activity is shown in **Figure 2D**. Recombinant strains were cultured in a 1-L shaker flask at 22°C, pH 6, and 1% (v/v) methanol. From 0 to 24 h, methanol was not added and no enzymatic activity was detected. With the first addition of methanol (24 h), methanol was used as the sole carbon source by the recombinant strains and κ-carrageenase secretion was observed. The maximum activity of the recombinant enzymes was detected at 96 h of incubation. The maximum activities for the recombinant enzymes cgkZ, cgkZΔPst, and cgkZΔCBM were 4.68, 5.70, and 3.02 U/mL, respectively.

The results for gene transcription levels as measured by qPCR are shown in **Table 3**. The transcription levels of *cgkZ*, *cgkZ*Δ*Pst*, and *cgkZ*Δ*CBM* were significantly higher at 36 h than at 72 h. Compared with the transcription level of *cgkZ*, the transcription levels of *cgkZ*Δ*Pst* and *cgkZ*Δ*CBM* increased to 2.9- and 3.5-fold at 36 h, and to 4.3- and 2.8-fold at 72 h, respectively. Since the gene copy numbers of *cgkZ* and *cgkZ*Δ*Pst* were similar, the higher enzymatic activity of cgkZΔPst was due to its higher transcription level. This result indicated that truncation of the Pst increased the expression of the recombinant enzyme. However, the enzymatic activity of cgkZΔCBM was depressed even though *cgkZ*Δ*CBM* showed a higher transcription level than *cgkZ*, indicating that the truncation of the CBM may decrease the enzymatic activity of κ-carrageenase.

**Table 3:** Determination of transcription levels by real-time qPCR.

cDNA	*C*_t_ value (GAPDH)	*C*_t_ value (target gene)	Transcript levels (related to GAPDH)
*cgkZ*(36 h)	15.39 ± 0.18	12.47 ± 0.49	14.39
*cgkZ*Δ*Pst*(36 h)	16.21 ± 0.42	11.97 ± 0.36	41.02
*cgkZ*Δ*CBM*(36 h)	16.38 ± 0.52	11.64 ± 0.61	50.39
*cgkZ*(72 h)	17.22 ± 0.58	15.32 ± 0.50	7.73
*cgkZ*Δ*Pst*(72 h)	18.63 ± 0.31	14.89 ± 0.36	33.96
*cgkZ*Δ*CBM*(72 h)	16.20 ± 0.07	12.82 ± 0.42	21.58

*C_*t*_ values are “mean ± standard deviation” from three replicates (*n* = 3)*.

### Purification of Recombinant κ-Carrageenase

**FIGURE 3:**
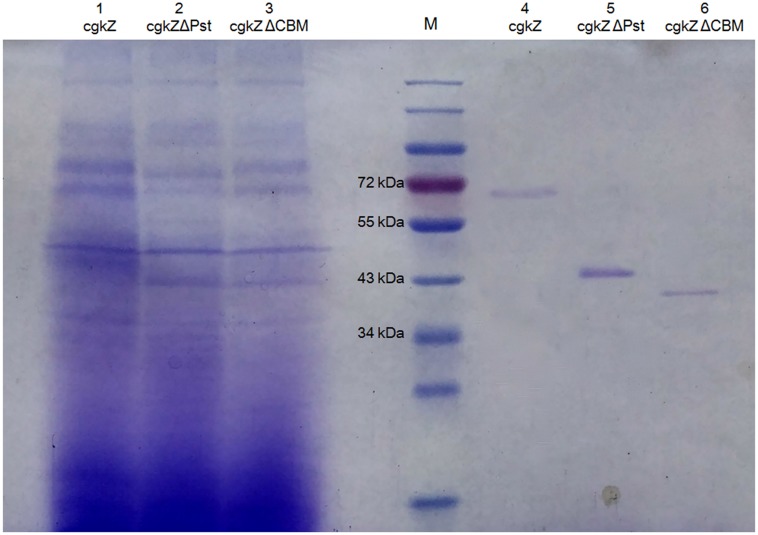
SDS-PAGE analysis of recombinant κ-carrageenase. Line M represents protein marker. Line 1–3 represents the supernatant of fermentation broth and Line 4–6 represents purified recombinant enzymes.

The supernatants obtained from cultures grown under shaker-flask fermentation were concentrated before cation exchange chromatography. As shown in **Table 4**, after cation exchange chromatography, the specific activity of cgkZ increased by 9.7-fold to 356.74 U/mg, while the specific activities of cgkZΔPst and cgkZΔCBM were 487.78 and 265.66 U/mg, representing 10.4- and 11.0-fold increases, respectively. The SDS-PAGE result of the purified recombinant κ-carrageenase is shown in **Figure 3**. The molecular weights of cgkZ, cgkZΔPst and cgkZΔCBM were found to be approximately 65, 45, and 40 kDa, respectively. The molecular weights of these proteins significantly differed and most of the impurities were removed after cation exchange chromatography.

**Table 4:** Purification and productivity of recombinant κ-carrageenase.

Name		Activity (U/mL)	Protein (μg/mL)	Specific activity (U/mg)	Activity recovery (%)	Purification (-fold)
cgkZ	Crude enzyme	4.68	127.14	36.82	100	1
	CM-Sepharose	5.95	16.68	356.74	35.9	9.7
cgkZΔPst	Crude enzyme	5.70	122.06	46.73	100	1
	CM-Sepharose	6.71	13.75	487.78	34.2	10.4
cgkZΔCBM	Crude enzyme	3.02	125.45	24.08	100	1
	CM-Sepharose	4.39	16.52	265.66	34.0	11.0

### Enzymatic Characterization of Recombinant κ-Carrageenase

**FIGURE 4:**
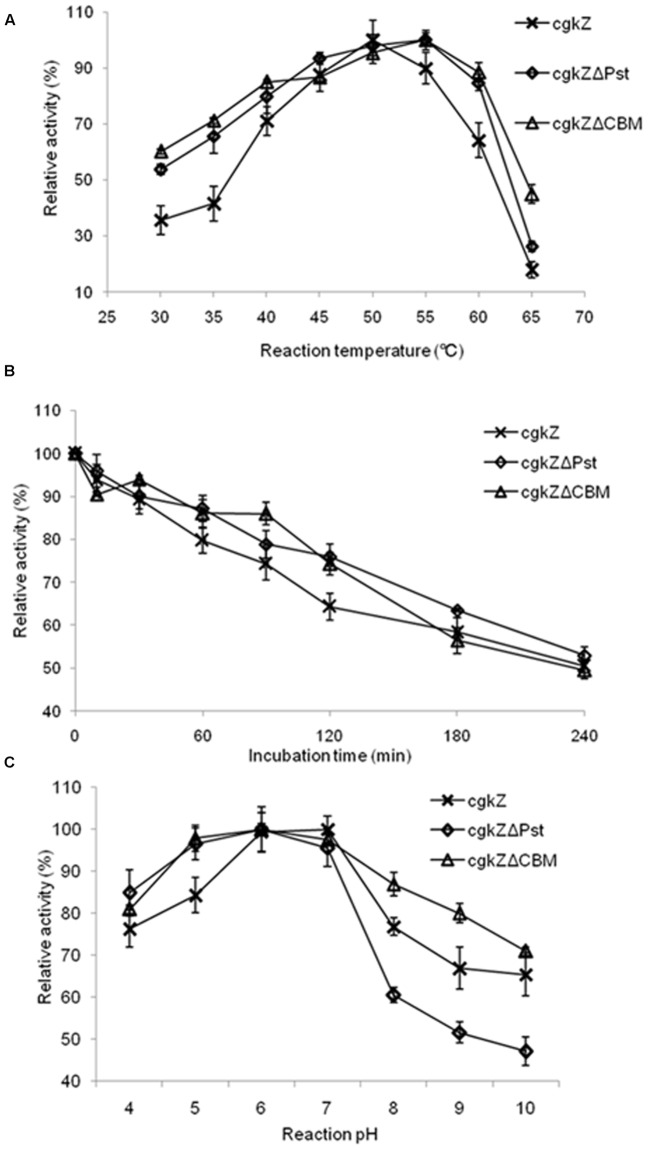
Characterization of recombinant κ-carrageenase. **(A)** The effect of temperature on enzyme activity. **(B)** The thermostability of recombinant κ-carrageenase. The incubation temperature is 45°C. **(C)** The effect of pH on enzyme activity. Error bars represent the standard deviation, *n* = 3.

In this study, the temperature and pH ranges selected for optimization experiments were chosen based on the enzymatic characterization of κ-carrageenase produced by *Zobellia* sp. ZM-2, which had an optimum temperature and pH of 39°C and 6.0, respectively (Liu et al., 2013). The effect of temperature on recombinant κ-carrageenase activity expressed in *P. pastoris* is shown in **Figure 4A**. The recombinant enzyme cgkZ showed the highest activity at 50°C and its relative activity decreased to 89% at 55°C. The recombinant enzymes cgkZΔPst and cgkZΔCBM showed the highest activities at 55°C and their relative activities decreased to 84 and 88% at 60°C, respectively.

In the experiment for the determination of the enzymes’ thermal stabilities, after incubation at 45°C for 4 h, approximately 50% of the enzymatic activity was retained (**Figure 4B**). When the recombinant enzymes were incubated at 42.5°C for 4 h, more than 90% of the enzymatic activity was retained (data not shown). All the recombinant enzymes were stable below 42.5°C. Approximately 50% of the enzymatic activity was lost after 1 h of incubation at 47.5°C and after 30 min of incubation at 50°C (data not shown). These results demonstrate that these recombinant enzymes expressed by *P. pastoris* have a higher thermal stability than one that was previously expressed by *E. coli*, which was stable below 35°C (Liu et al., 2013). κ-Carrageenase obtained from *Pseudoalteromonas porphyrae* showed thermal stability at 30°C (Liu et al., 2011), which was also lower than the thermal stabilities of the recombinant enzymes expressed in this study.

The effect of pH on recombinant κ-carrageenase activity is shown in **Figure 4C**. The recombinant enzyme cgkZ showed the highest activity at pH 7 and its optimum pH was 6–7. The recombinant enzymes cgkZΔPst and cgkZΔCBM showed the highest activities at pH 6.0, with an optimum pH of 5–7. For recombinant enzyme cgkZ, at pH 4 or 8, the relative activity decreased to approximately 76%, indicating that it began to become inactivated under those conditions.

The effects of ions and chemical reagents on recombinant κ-carrageenase activity are shown in **Table 5**. The results show that Na^+^ promoted the activity of recombinant κ-carrageenase, while a high concentration of K^+^ inhibited enzymatic activity. Mg^2+^ and Ca^2+^ stimulated the enzymatic activity slightly, while Cu^2+^, Zn^2+^, Fe^2+^, and Fe^3+^ strongly inhibited the activity. Among the tested chemical reagents, the addition of DTT stimulated enzymatic activity to 178–217%, which was similar to the reports of Yao et al. (2012) and Liu et al. (2013). SDS significantly inhibited the enzymes. EDTA had slight inhibitory effects on the recombinant enzymes. TritonX-100, Tween-80, methanol, and glycerol had minimal effects on the activities of the three recombinant enzymes.

**Table 5:** Effects of ions and chemical reagents on the activity of recombinant κ-carrageenase.

Ions and chemical reagents	Concentrations	Relative activity of cgkZ (%)	Relative activity of cgkZΔPst (%)	Relative activity of cgkZΔCBM (%)
Na^+^	10 mM	107.8 ± 3.75	108.0 ± 5.25	97.0 ± 1.51
Na^+^	50 mM	117.3 ± 1.28	123.7 ± 1.35	122.8 ± 1.23
Na^+^	100 mM	129.1 ± 1.88	130.7 ± 4.90	119.2 ± 1.49
K^+^	10 mM	118.5 ± 4.21	109.8 ± 1.98	101.2 ± 0.38
K^+^	50 mM	49.4 ± 1.78	68.6 ± 2.31	98.3 ± 1.22
K^+^	100 mM	34.6 ± 1.86	38.1 ± 1.85	10.6 ± 0.15
Cu^2+^	5 mM	20.2 ± 2.49	25.0 ± 0.10	21.7 ± 0.25
Ca^2+^	5 mM	101.5 ± 3.07	115.4 ± 1.28	104.1 ± 1.17
Zn^2+^	5 mM	28.0 ± 0.59	54.6 ± 4.20	58.4 ± 0.89
Mg^2+^	5 mM	104.3 ± 0.91	110.7 ± 1.86	106.9 ± 1.84
Fe^2+^	5 mM	68.2 ± 2.53	76.7 ± 1.55	79.5 ± 1.34
Fe^3+^	5 mM	24.7 ± 2.40	35.9 ± 3.35	31.0 ± 0.55
DTT	5 mM	177.8 ± 2.68	216.8 ± 8.61	193.6 ± 2.25
EDTA	5 mM	98.4 ± 2.08	84.9 ± 5.34	22.3 ± 0.13
SDS	5 mM	16.2 ± 2.09	27.7 ± 4.38	17.7 ± 0.25
Triton-100	0.5% (v/v)	101.9 ± 3.08	103.8 ± 3.89	103.0 ± 1.67
Tween-80	0.5% (v/v)	106.7 ± 0.31	101.6 ± 2.27	101.4 ± 0.54
Methanol	0.5% (v/v)	101.2 ± 2.31	108.6 ± 5.51	105.3 ± 2.68
Glycerol	0.5% (v/v)	103.2 ± 0.87	102.6 ± 4.45	102.9 ± 1.15

*Values are “mean ± standard deviation” from three replicates (*n* = 3)*.

Based on the Lineweaver–Burk double reciprocal plots, the K_m_ values of cgkZ, cgkZΔPst and cgkZΔCBM were 2.07, 1.85, and 1.04 mg/mL, respectively, which were higher than that reported by Liu et al. (2013) (0.84 mg/mL). Compared with the κ-carrageenase obtained from *P. porphyrae* (K_m_ 4.4 mg/mL), these recombinant enzymes showed higher substrate affinities (Liu et al., 2011). The V_max_ values of these recombinant enzymes were 0.77, 1.35, and 0.50 mg/mL⋅min, respectively.

### Analysis of Recombinant Enzymatic Hydrolysis Products

**FIGURE 5:**
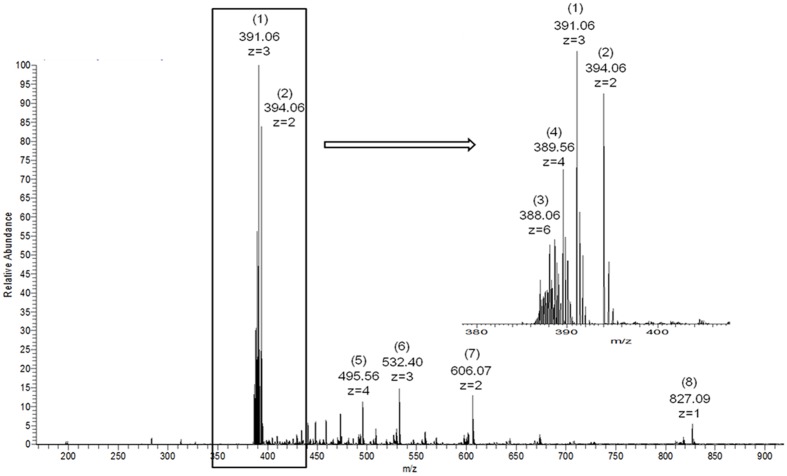
Electrospray ionization mass spectrometry spectra of recombinant κ-carrageenase hydrolysis products. (1) [A-G_4S_]_3_, (2) [A-G_4S_]_2_, (3) [A-G_4S_]_6_, (4) [A-G_4S_]_4_, (5) [A-G_4S_]_5_+K, (6) [A-G_4S_]_4_+K, (7) [A-G_4S_]_3_+K, (8) [A-G_4S_]_2_+K. A represents (3, 6)-anhydrogalactose, G_4S_ represents 4-sulfated galactose, [A-G_4S_] represents the repeating unit of κ-carrageenan and K represents potassium.

Although the recombinant enzymes were encoded by target genes with different lengths, the end-products after the enzymatic hydrolysis of κ-carrageenan were nearly the same. The MS spectrum is shown in **Figure 5**. Two intense fragment peaks were observed. An ion at *m/z* 394 (*z* = 2) with a molecular weight of 790 Da represented a tetrasaccharide with two [A-G_4S_] repeating units ([A-G_4S_]_2_). Another ion at *m/z* 391 (*z* = 3) with a molecular weight of 1176 Da represented a hexasaccharide with three [A-G_4S_] repeating units ([A-G_4S_]_3_) (Yu et al., 2017). Some other weak peaks were also observed. The results of MS showed that the final products of enzymes cgkZ, cgkZΔPst and cgkZΔCBM-catalyzed hydrolysis were mainly composed of κ-carrageenan tetrasaccharide and hexasaccharide, which is similar to the results obtained by Liu et al. (2013). Although the Pst and CBM were truncated in cgkZΔPst and cgkZΔCBM, respectively, the catalytic properties of these enzymes did not change compared with those of the full-length recombinant enzyme.

## Discussion

The full-length *cgkZ* and its truncated gene (*cgkZ*Δ*Pst, cgkZΔCBM*) were expressed in *P. pastoris*. The extracellular enzymatic activities are 4.68, 5.70, and 3.02 U/mL, respectively. The difference in their molecular weights (65, 45, and 40 kDa, respectively) demonstrates the success of gene truncation. The three recombinant enzymes showed some common characteristics. Firstly, their enzymatic activities increased to 178–217% in the presence of the reducing agent DTT, and some metal ions (Cu^2+^, Zn^2+^, and Fe^3+^) strongly inhibited their activities. Recombinant cgkZ has four cysteines (Cys), among which Cys137 is located in the active site of κ-carrageenase (Liu et al., 2013). It was reported that heavy metal ions have strong affinities for sulphydryl (-SH) residues, therefore, the presence of heavy metal ions results in the negative conformational changes of recombinant enzymes (Yao et al., 2012; Liu et al., 2013). DTT can prohibit the formation of disulfide bonds and maintaining the regular conformational folding of enzyme. The catalytic Cys should be maintained in an active state under the protection of DTT (Zhu et al., 2011; Yao et al., 2012). Therefore, DTT is used as an enzymatic stimulation reagent in some cases.

Secondly, all these recombinant enzymes showed optimal temperature exceeding 45°C, and more than 60% of their activities were retained after 3 h of incubation at 45°C. For comparison, the optimum temperature of cgkZ expressed in *E. coli* was 39°C (Liu et al., 2013). The thermal stability of κ-carrageenase expressed in *P. pastoris* was at least 5°C higher than those of the same protein expressed in *E. coli*. It was previously reported that *N*-glycosylation can significantly improve the thermal stability of recombinant enzymes expressed using *P. pastoris* (Guo et al., 2008; Han et al., 2014; Zhao et al., 2015). *N*-Glycosylation generates hydrogen bond networks surrounding the recombinant enzymes and enhances their conformational stability (Zou et al., 2013; Zhao et al., 2015). *N*-Glycosylation also significantly decreases dynamic fluctuations in the molecule structure of enzymes, which helps to improve their thermal stabilities (Yang et al., 2016). In proteins expressed using *P. pastoris*, *N*-glycosylation is mainly found in recombinant proteins containing the Asn-X-Ser/Thr sequence. In the present study, analyses using the NetNGlyc 1.0 server database^2^ revealed Asn70 and Asn255 as potential *N*-glycosylation sites in recombinant κ-carrageenase. It was reported that in *P. pastoris* expressed proteins, the *N*-glycosylation occupied 21% (recombinant neutral protease I) and 30% (recombinant lipase A) of the total molecular weight, respectively (Lei et al., 2013; Vici et al., 2015). The molecular weight of cgkZ expressed in *P. pastoris* was approximately 65 kDa, which was higher than that of the same protein expressed in *E. coli* (45 kDa, Liu et al., 2013). These results suggest that *N*-glycosylation may have been present in the recombinant enzymes and thus promotes the increase of thermal stabilities.

Thirdly, the hydrolysis products obtained by catalysis mediated by recombinant cgkZ expressed in *P. pastoris* and *E. coli* were similar and were mainly composed of κ-carrageenan tetrasaccharide and hexasaccharide. The conserved sequence of the catalytic domain did not change after the truncation of the Pst and CBM of κ-carrageenase. Thus, the enzymatic properties and catalytic products remained unchanged.

The three recombinant enzymes showed significant differences in their enzymatic activities and gene transcription levels. κ-Carrageenase belongs to glycoside hydrolase family 16 (GH-16). From the description of the full-length κ-carrageenase sequence in the NCBI GenBank database^3^, the CBM is located behind the catalytic domain, while the Pst is located at the C-terminal. The Pst was usually found at the C-terminal of proteins from gram-negative strains such as *Zobellia* sp. and *Porphyromonas* sp., and it promotes the secretion of proteins (Sato, 2011; Saiki and Konishi, 2012, 2014). The secretion vector pPIC9K used in *P. pastoris* expression system contains sequence encoding the α-factor signal peptide. Thus, the recombinant enzymes were secreted extracellularly and could be obtained by centrifugation. Therefore, the C-terminal Por secretion tail of the κ-carrageenase gene is not necessary in *P. pastoris* expression systems. It was reported that the truncation of several C-terminal amino acids from a recombinant xylanase belonging to the GH-10 family did not significantly affect its structure and function. Compared with the full-length xylanase, the specific activity of the truncated variant was improved by approximately 1.3-fold (Zheng et al., 2016). The target genes *cgkZ* and *cgkZ*Δ*Pst* had the same copy numbers. It was reported that gene copy number and heterologous protein expression had a linear correlation (Parashar and Satyanarayana, 2017). From the results of shaker-flask fermentation and purification, cgkZΔPst showed a 1.2-fold higher enzymatic activity and 1.4-fold higher specific activity than cgkZ. Meanwhile, the transcription levels of *cgkZ*Δ*Pst* were also improved compared with that of *cgkZ*. These results showed that the Pst-truncated enzyme had a higher activity owing to a higher transcription level. None of negative effects on enzymatic properties were detected after Pst truncation.

The CBM of κ-carrageenase has a β-sandwich fold structure with a single carbohydrate binding site on the surface of the enzyme (Ribeiro et al., 2010). Although the CBM is a non-catalytic module which is independent from the catalytic domain, its function of substrate targeting contributes to the improvement of the enzymatic activity and substrate affinity (Grondin et al., 2014; Verma et al., 2015; Mompeán et al., 2017). The modification or removal of the CBM may significantly reduce the hydrolytic activity toward insoluble substrates and can sometimes affect the enzymatic activity toward soluble substrates (Mizutani et al., 2014; Kim et al., 2016). For instance, the truncation of the CBM 22 domain of xylanase decreased its relative activity toward insoluble xylan to 75%, and the substrate affinity decreased to 80 % in comparison with the full-length xylanase (Mollerup et al., 2016; Sermsathanaswadi et al., 2017). The enzymatic activity and specific activities of cgkZΔCBM were 68 and 74% lower than those of cgkZ, respectively. Although the CBM truncated enzyme showed a higher transcription level than the full-length cgkZ, its enzymatic activity significantly decreased owing to a lowered efficiency of substrate binding. It is predicted that the CBM has an important role in maintaining the enzymatic activity of κ-carrageenase.

## Conclusion

The full-length and truncated recombinant κ-carrageenase enzymes expressed in *P. pastoris* showed superior thermal stabilities compared with the same enzymes expressed in *E. coli*. The Pst-truncated enzyme showed a higher transcription level and enzymatic activity than the full-length enzyme. The CBM-truncated enzyme showed a significant loss of enzymatic activity. The three recombinant enzymes possessed similar enzymatic properties. The application of the *P. pastoris* expression system combined with rational gene modification may be useful for preparing recombinant enzymes with high activities. This approach also provides a theoretical basis for the preparation of other marine glycoside hydrolase enzymes.

## Author Contributions

YY performed all the experiments, coordinated the data analysis, and prepared this manuscript. ZL contributed in the experiment skills education. HM and LL contributed in the experimental proposal and manuscript polishing. MY, MC, ZW, and LS provided part of research work suggestion, prepared part of research materials and supervised this study.

## Conflict of Interest Statement

The authors declare that the research was conducted in the absence of any commercial or financial relationships that could be construed as a potential conflict of interest.

## Footnotes

1 https://blast.ncbi.nlm.nih.gov/Blast.cgi

2 http://www.cbs.dtu.dk/services/NetNGlyc/

3 https://blast.ncbi.nlm.nih.gov/Blast.cgi
